# Protease nexin-1 deficiency increases mouse hindlimb neovascularisation following ischemia and accelerates femoral artery perfusion

**DOI:** 10.1038/s41598-021-92794-9

**Published:** 2021-06-28

**Authors:** Sonia Selbonne, Celina Madjene, Benjamin Salmon, Yacine Boulaftali, Marie-Christine Bouton, Véronique Arocas

**Affiliations:** 1 LVTS, INSERM, U1148, Paris, France; 2 Université de Paris, Paris, France; 3 EA 2496 Pathologies Imagerie Et Biothérapies de L’organe Dentaire, Faculté de Chirurgie Dentaire, Universitéde Paris, Montrouge, France; 4 Unité INSERM U1148, CHU Xavier Bichat, 46 rue Henri Huchard, 75877 Paris Cedex 18, France

**Keywords:** Peripheral vascular disease, Angiogenesis, Angiogenesis

## Abstract

We previously identified the inhibitory serpin protease nexin-1 (PN-1) as an important player of the angiogenic balance with anti-angiogenic activity in physiological conditions. In the present study, we aimed to determine the role of PN-1 on pathological angiogenesis and particularly in response to ischemia, in the mouse model induced by femoral artery ligation. In wild-type (WT) muscle, we observed an upregulation of PN-1 mRNA and protein after ischemia. Angiography analysis showed that femoral artery perfusion was more rapidly restored in PN-1^−/−^ mice than in WT mice. Moreover, immunohistochemistry showed that capillary density increased following ischemia to a greater extent in PN-1^−/−^ than in WT muscles. Moreover, leukocyte recruitment and IL-6 and MCP-1 levels were also increased in PN-1^−/−^ mice compared to WT after ischemia. This increase was accompanied by a higher overexpression of the growth factor midkine, known to promote leukocyte trafficking and to modulate expression of proinflammatory cytokines. Our results thus suggest that the higher expression of midkine observed in PN-1- deficient mice can increase leukocyte recruitment in response to higher levels of MCP-1, finally driving neoangiogenesis. Thus, PN-1 can limit neovascularisation in pathological conditions, including post-ischemic reperfusion of the lower limbs.

## Introduction

Peripheral artery disease (PAD) is an atherosclerotic occlusive disease of the lower extremities associated with pain, limited mobility, and an elevated risk of amputation. PAD is characterized by skeletal muscle ischemia without induction of sufficient angiogenesis to restore normal perfusion. The main treatment for limiting claudication and critical limb ischemia is revascularization, either surgical or endovascular. However, some patients are not anatomically amenable to revascularization or have suffered failed revascularization. New therapies are therefore still needed. In contrast to promising results from animal studies, administration of proangiogenic factors such as fibroblast growth factor (FGF) or vascular endothelial growth factor (VEGF) failed to induce significant improvement in ischemia in several phase 1 clinical trials^1^, making research into new strategies necessary.

Many factors are involved in angiogenesis, including growth factors, chemokines, and proteases that play different roles in promoting and conducting tissue regeneration. The protease/anti-protease balance plays a crucial role in the completion of an appropriate and functional vascular network. Among the protease inhibitors, some serpins (serine protease inhibitor) are known to have a pro- or anti-angiogenic activity and have been implicated in ischemia-induced neoangiogenesis^2–4^. Proteins belonging to the serpin superfamily play central regulatory roles throughout the mammalian body, serving as pivotal points in normal physiological functions. Most serpins are powerful specific inhibitors of extracellular serine proteases controlling biological processes such as blood coagulation, fibrinolysis, tissue remodelling, and inflammation. Their role as central regulators of normal physiological pathways is well illustrated by disease states due to serpin deficiency in humans and by the profound effects induced by genetic mutations in some serpins. The inhibitory serpin protease nexin-1 (PN-1), or serpin E2, is expressed in the vascular wall, and is stored in platelets from which it is secreted upon activation^5^. Through its anti-thrombotic and anti-fibrinolytic activities, PN-1 is a key regulator of vascular responses to injury^6^ and is overexpressed in numerous diseases where an important inflammatory reaction takes place, like atherosclerosis^7,8^, scleroderma^9^, or idiopathic pulmonary fibrosis (IPF)^10^. In addition, we have shown that PN-1 is also an important actor of the angiogenic balance. PN-1 is indeed a negative regulator of retinal vascular development^11^. PN-1-deficient mice exhibit thicker and denser capillaries, and an increased number of veins and arteries, compared to wild-type (WT) mice. In *vitro*, PN-1 inhibits the proliferation, migration, capillary tube formation and spreading of endothelial cells in response to VEGF^12^. The in vitro anti-angiogenic effect of PN- 1 is independent of its anti-protease activity but depends partly on its binding to cell surface glycosaminoglycans^12^. I*n vivo*, we showed that it is independent of VEGF expression, but is associated with a modified expression of the growth factor, midkine (MK)^11^. MK is a heparin-binding growth factor, and has been implicated in neuronal survival and differentiation, cancer development and inflammation-related diseases^13^. It has been shown to promote angiogenesis both in tumours^14^ and in response to ischemia^15^. In addition, MK supports neutrophil trafficking during acute inflammation by promoting adhesion via β2 integrins (CD11/CD18)^16^. In patients with critical limb ischemia, a higher neutrophil count may predict limb loss within 6 months. Intermittent claudication is associated with increased activation of neutrophils and release of mediators that may further impair endothelial function.

In this study, we aimed to determine the role of PN-1 on pathological angiogenesis and particularly in response to ischemia. As PN-1 strongly limits physiological angiogenesis and is expressed by inflammatory cells^7^, we postulate that it could modulate PAD outcome. In this context, we used a mouse model of hindlimb ischemia induced by ligation of the femoral artery. We analysed PN-1 expression kinetics in response to ischemia and the effect of PN-1 deficiency on muscle vasculature in ischemic conditions. Finally, we analysed the level of the inflammatory reaction in muscles and the expression of factors whose link to PN-1 was identified in previous studies^11^. We clearly show that PN-1 modulates post-ischemic neo-angiogenesis with modifications of MK expression and of leukocyte recruitment. This study thus identifies PN-1 as a possible target to improve muscle perfusion in PAD.

## Results

We used the mouse model of hindlimb ischemia induced by femoral artery ligation in WT and PN-1^−/−^ mice and analysed muscles after 3, 5, 7 and 14 days. This model is a common model to study PAD and allows observation of the neovascularization occurring to rescue flow, and thus to irrigate the distal limb. In mice, a new capillary network is formed by angiogenesis downstream to the ligature whereas collateralization occurs in the portion proximal to the ligation^17^.

### PN-1 expression is increased in hindlimb muscle following ischemia

**Figure 1:**
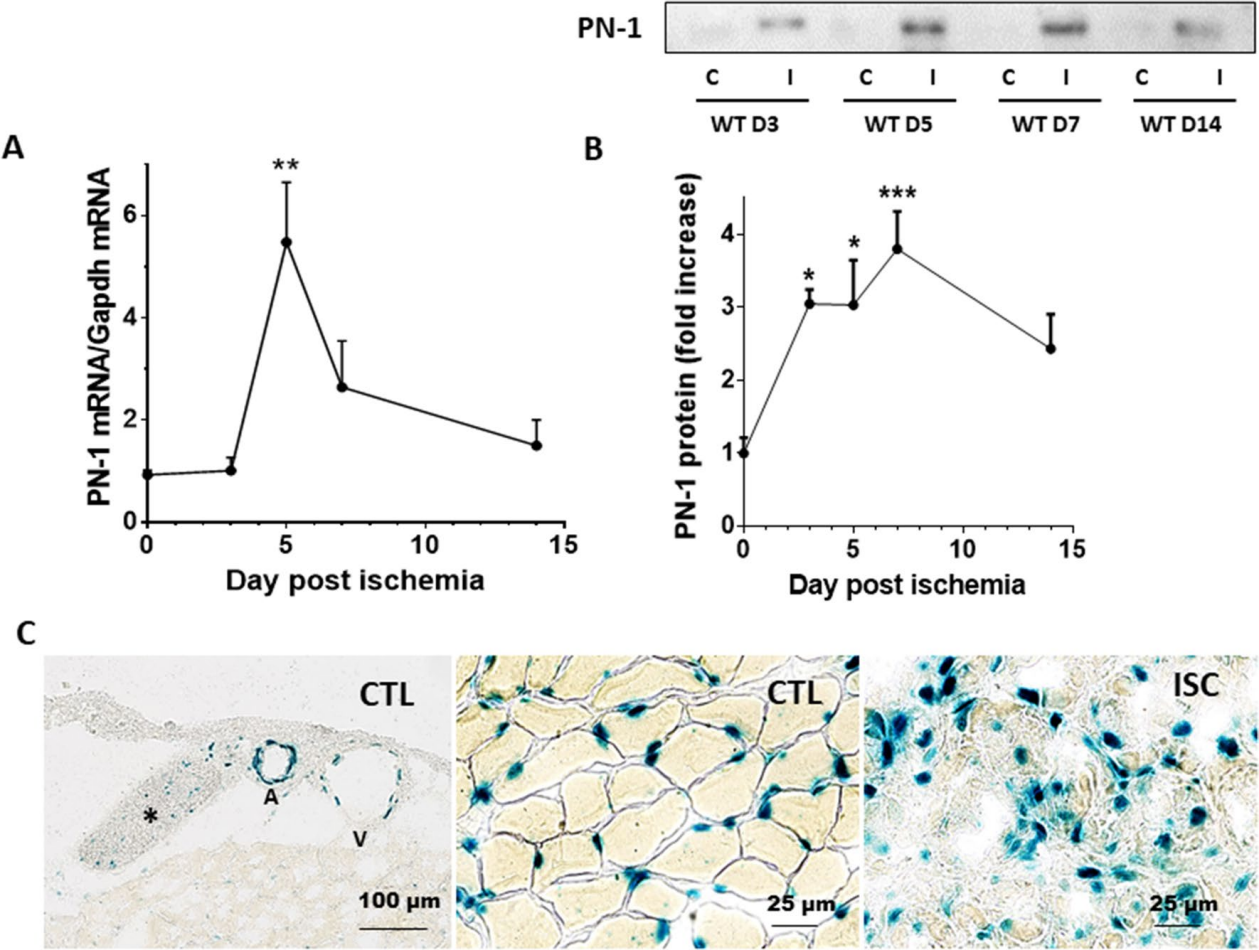
PN-1 muscle expression increases following ischemia. (**A**) Quantification of PN-1 mRNA by qRT-PCR; Anova **p < 0.01; (**B**) Immunoblotting of muscle lysates with antibody to PN-1 and densitometric analysis; Anova *p < 0.05, ***p < 0.001. The uncropped image is shown in supplementary Fig. S2, (**C**): XGal staining on frozen muscle sections from PN-1/LacZ mice, representative images out of 4; first panel: control muscle * = nerve fibre, A: artery, V: vein; second panel: magnification of control gastrocnemius; third panel: magnification of ischemic gastrocnemius, 7 days after ligation.

We first analysed PN-1 expression in mouse tibialis ischemic muscles. RT PCR analysis indicated a 5.7-fold increase in PN-1 mRNA, 5 days after ischemia, followed by a progressive decrease up to day 14 (Fig. 1A). PN-1 protein, analysed by western blotting, was only weakly detected in normal muscle. However, the intensity of the band corresponding to PN-1 transiently increased following ischemia with a maximum at days 5–7 (Fig. 1B), demonstrating a significant upregulation of PN-1 following ischemia. X-gal staining of control muscles from PN-1/LacZ mice, in which beta galactosidase expression is under PN-1 promoter and directed to the nucleus, indicated that PN-1 expression in intact muscle architecture was detected in nerve fibres, arteries, veins as well as myofibres and capillaries. Following ischemia, we observed that PN-1 was also expressed in the infiltrating inflammatory cells, as illustrated by the X-gal staining in the damaged muscle (Fig. 1C).

### Femoral artery reperfusion is faster in PN-1-deficient mice

**Figure 2:**
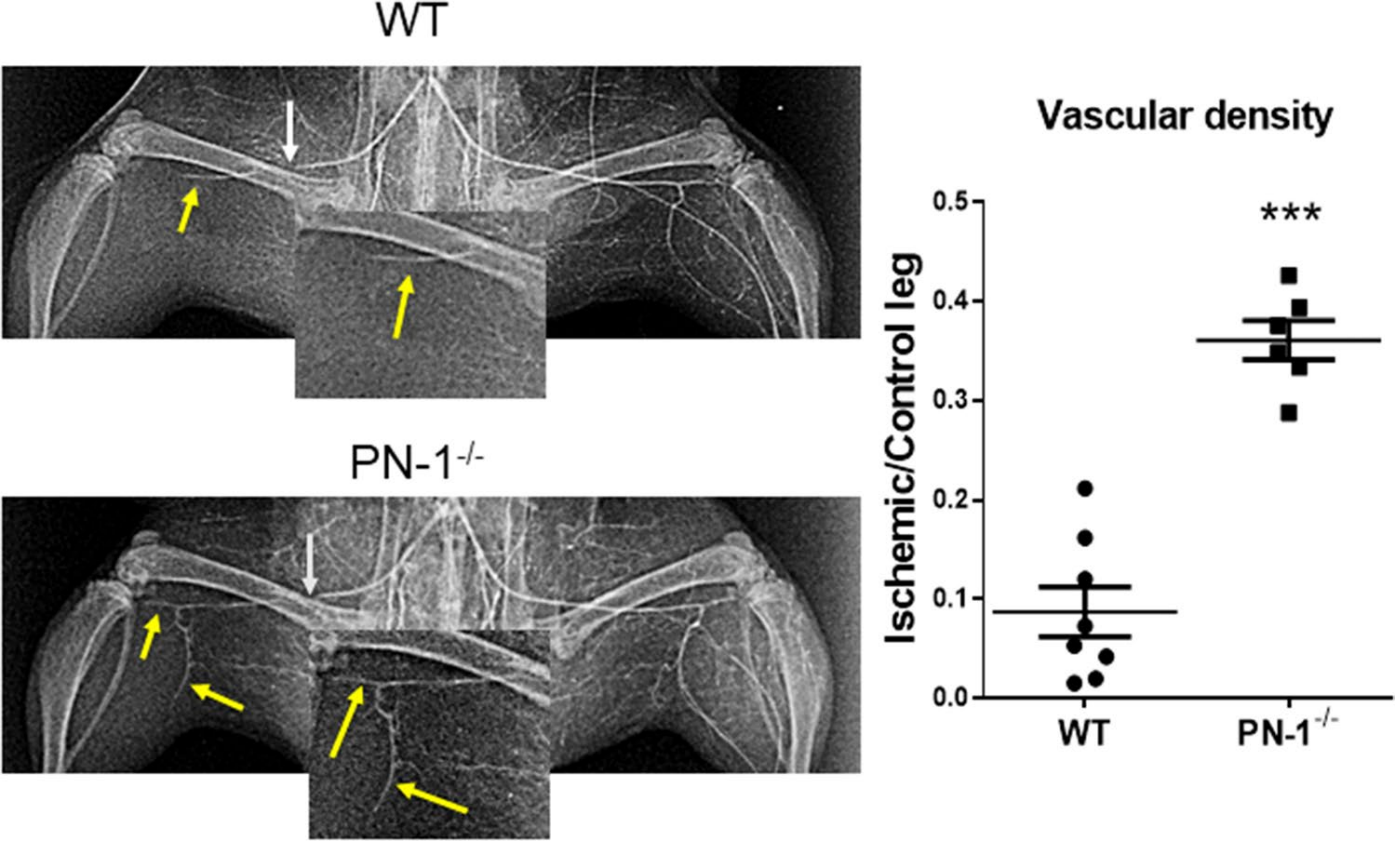
Representative photomicrographs and quantitative evaluation of microangiography. White arrows indicate the ligation, yellow arrows the reperfused vessels. Vascular density was determined by measuring the number of pixels occupied by vessels. t test ***p < 0.001.

The above results led us to examine the effect of PN-1 deficiency on the hindlimb vascularization and post-ischemic neovascularization in mice. We thus performed angiography to analyse blood perfusion in the limb following ischemia. Previous studies^17,18^ and analyses at different times following ischemia indicated that revascularization occurred rapidly in WT C57BL/6 mice in our conditions. We thus compared PN-1^−/−^ and WT mice as early as day 3 following ischemia (Fig. 2). Angiographic images indicated that femoral artery perfusion was already partially restored in PN-1^−/−^ mice whereas this was not the case in WT mice. Quantification showed a significant (4.1-fold) increase in the angiographic score (Vessel density Ischemic/Non-Ischemic limb) in PN-1-deficient mice compared to control mice (*p* < 0.0001). These results thus indicate that PN-1 deficiency favours muscle reperfusion.

### Neovascularization is enhanced in PN-1-deficient mice

**Figure 3:**
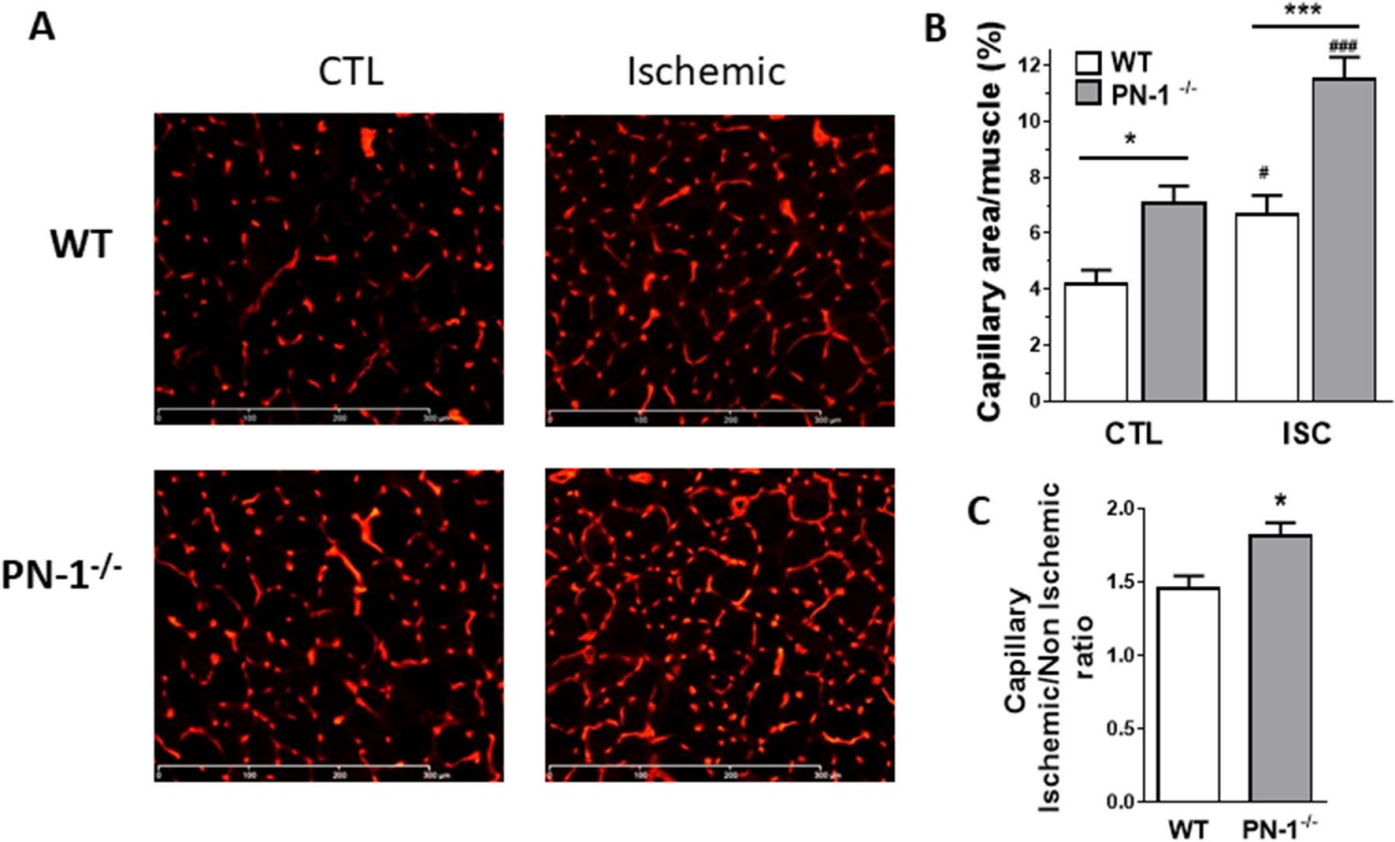
PN-1 deficiency results in higher capillary density in normal and ischemic muscle. (**A**) Capillaries were visualized by Isolectin B4 staining of control (CTL) and ischemic muscles from WT and PN-1^−/−^ mice 7 days after surgery. Representative images are shown. (**B**) Capillary density was quantified as the percentage of area covered by vessels (n ≥ 7). Statistical analysis was performed using Anova. (**C**) Ratio of capillary density of ischemic to non-ischemic muscles was calculated for each mouse (n = 7); t test: *p < 0.05; *** or ###*p* < 0.001; *: KO versus WT and #: ischemic versus control.

We thus measured muscle capillary density by analysing isolectin B4 labelling on frozen muscle sections. In agreement with the results previously observed^11^, capillary density was increased 1.5-fold in non-ischemic PN-1^−/−^ muscle compared to WT muscle (Fig. 3A,B, CTL). Hindlimb ischemia induced an increase in capillary density in both WT and PN-1^−/−^ muscles. However, it is noteworthy that ischemic over non-ischemic density ratio quantified at day 7 (Fig. 3A,B, ISC) was significantly higher in PN-1^−/−^ than in WT ischemic muscles (Fig. 3C,* p* = 0.0146), reflecting increased neoangiogenesis in PN-1^−/−^ mice.

**Figure 4:**
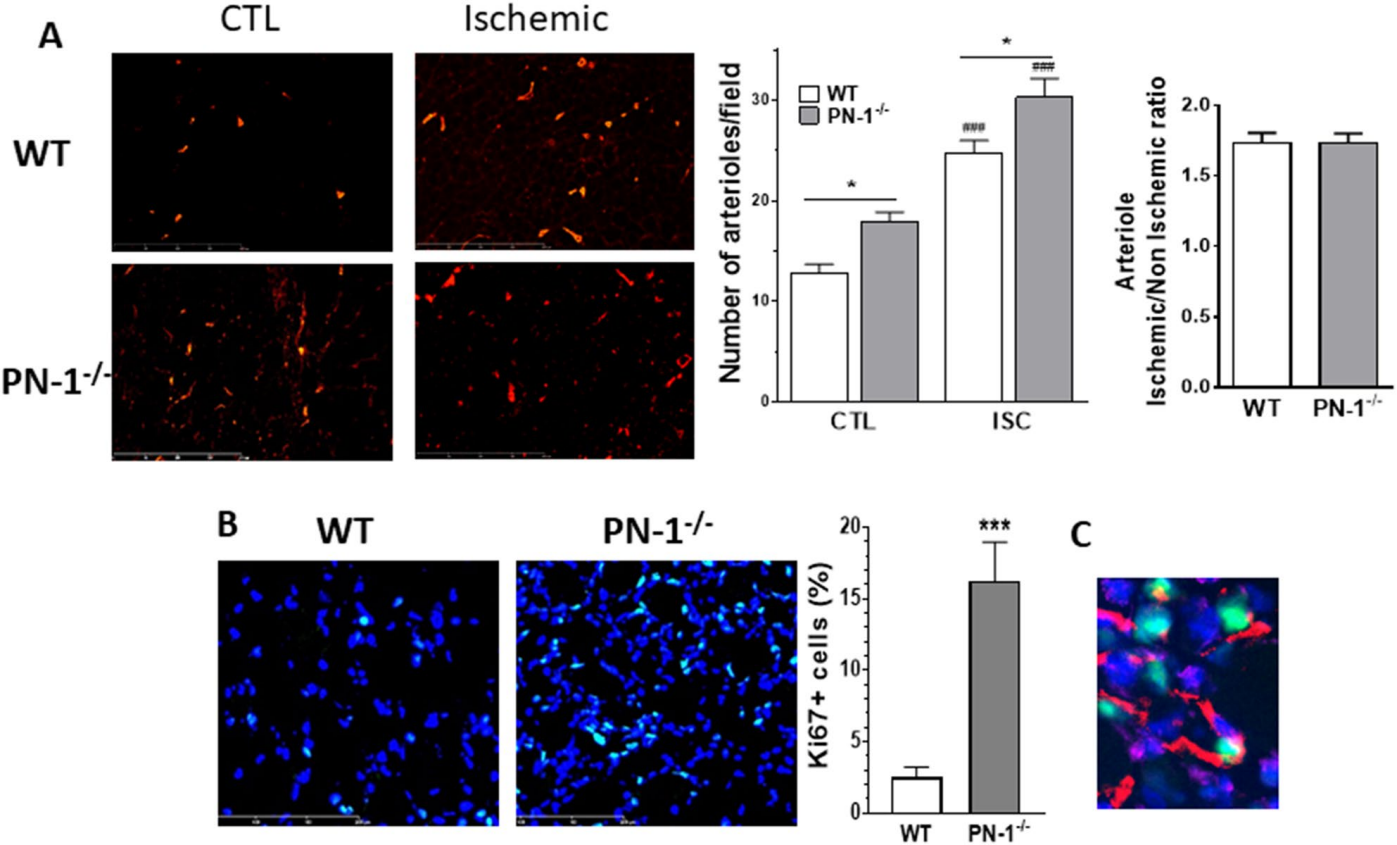
PN-1 deficiency does not modify arteriogenesis but results in higher arterial count and greater proliferation. (**A**) Representative images of α smooth muscle actin labelling of control (CTL) and ischemic (ISC) muscles sections. Arterioles were manually counted and statistical analysis was performed with Anova test (n > 10), (**B**) Representative images of Ki67 labelling (green, DAPI in blue). No positive nuclei were observed in control muscles. Quantification was performed by counting Ki67-postive nuclei and expressed as the percentage of total nuclei. (**C**) Representative image of co-labelling of Ki67 (green) and VE Cadherin (red) on ischemic muscle section. Blue: DAPI. Statistical analysis performed with t test (n = 9): *p < 0.05; *** or ###*p* < 0.001; *: KO versus WT and #: ischemic versus control.

We also observed an increased number of arterioles, counted on muscle sections after αSMA labelling, in non-ischemic PN-1^−/−^ muscle compared to WT (1.3-fold). This number also increased following ischemia in both types of mice but in contrast to capillary density, the ischemic over non-ischemic arteriole density ratio was similar in WT and PN-1^−/−^ muscles (1.8 and 1.7-fold, respectively, Fig. 4A).

To determine if the increased neoangiogenesis observed in PN-1^−/−^ ischemic muscles was related to an increase in endothelial cell proliferation, we analysed the labelling of the proliferation marker, Ki67. No labelling was observed in control muscles. In contrast, we observed a significantly higher (6.7-fold) number of proliferating cells in PN-1^−/−^- compared to WT- ischemic muscles (Fig. 4B), in agreement with the enhanced neovascularisation observed in PN-1^−/−^ ischemic muscles. Co-labelling with anti VE-Cadherin confirmed that most of these proliferating cells were endothelial cells (Fig. 4C).

### PN-1 deficiency is associated with increased expression of midkine

**Figure 5:**
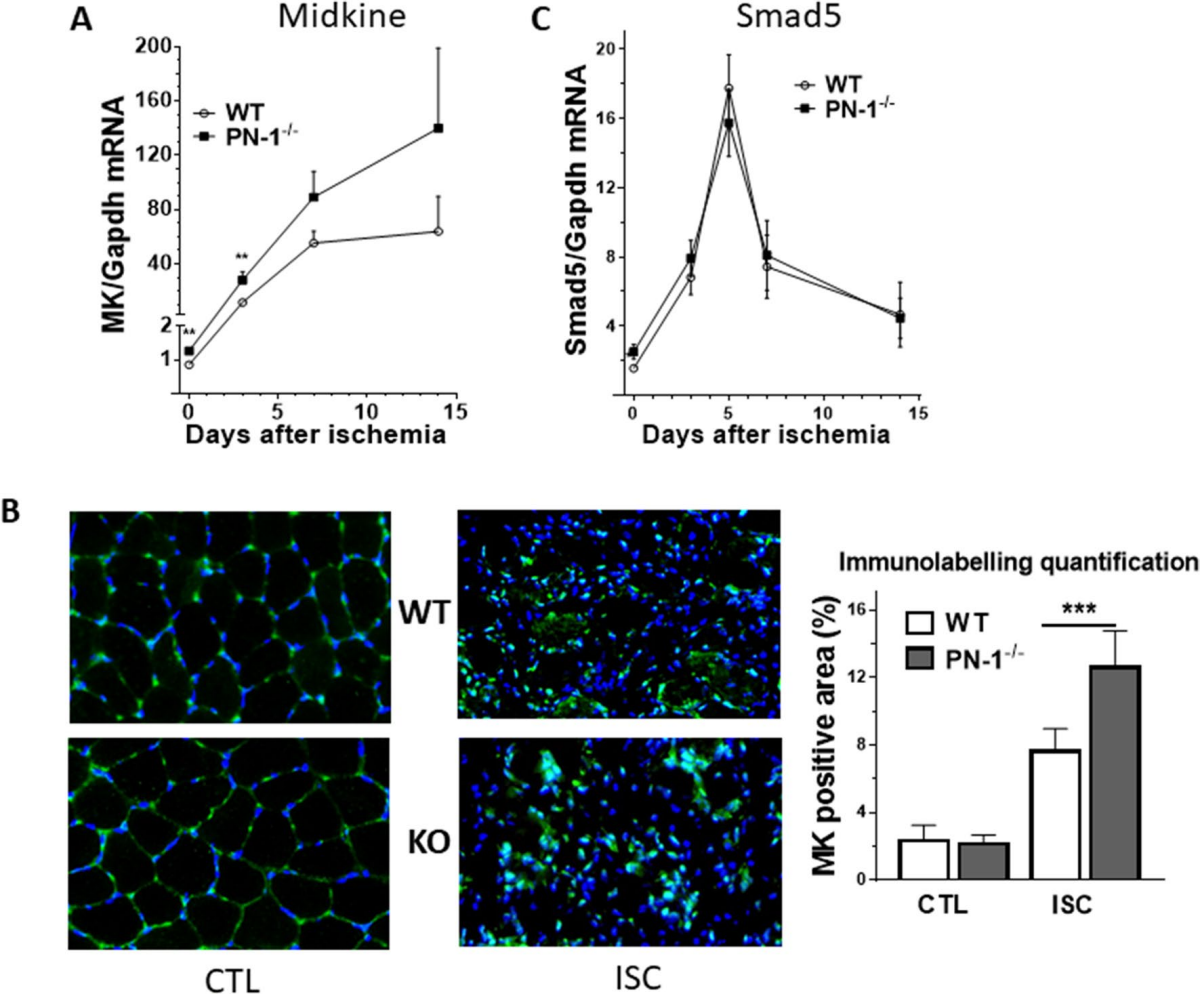
Midkine expression level is increased in PN-1-deficient mice, in control conditions and after ischemia, (**A**) Expression kinetics of Midkine mRNA following ischemia; Mann Whitney analysis. (**B**) Midkine immunolabelling (midkine in green, DAPI in blue) of gastrocnemius frozen sections (CTL) and 3 days after ischemia (ISC). Mann Whitney **p < 0.01. Representative images and quantification are shown (n = 4). Anova, ***p < 0.001. (**C**) Expression kinetics of Smad5 mRNA following ischemia.

We previously identified MK and Smad5 as genes whose expression was modified during physiological angiogenesis in PN-1-deficient retina compared to WT. We thus compared their expression in control and ischemic muscles from PN-1^−/−^ and WT mice. MK expression in non-ischemic muscle was very low but significantly higher in PN-1^−/−^ compared to WT (1.6-fold, Fig. 5A, day 0) in agreement with our previous results^11^. Following ischemia, we observed that MK expression rapidly increased in WT mice to reach a 60-fold increase between day 5 and 7 and remained elevated up to day 14 (Fig. 5A), in agreement with results of Weckbach et al.^15^ that showed an increased expression of MK by HUVECs following hypoxia. MK overexpression induced by ischemia was 2.2-fold higher in PN-1-deficient mice than in WT mice at day 3 (Fig. 5A), and remained higher up to day 14, although the difference with WT was non-significant (Fig. 5A).

These results were confirmed by immunohistochemical analysis of MK protein (Fig. 5B). No difference was evidenced in control conditions, whereas a significant increase in MK labelling was observed after ischemia in PN-1-deficient muscles compared to WT (Fig. 5B). Our results thus indicate that PN-1 deficiency is associated with an increase in MK expression following ischemia. Smad5 expression was also significantly higher in PN-1^−/−^ compared to WT in non-ischemic muscle (1.36-fold, Fig. 5C) as previously described^11^. Smad5 mRNA levels also increased following ischemia with a peak at day 5 (9.2-fold compared to control muscle) and then rapidly decreased to lower levels (threefold the control value at day 14). However, the Smad5 expression profile in PN-1 and WT deficient muscles was similar.

Other members of the Smad family proteins susceptible to be associated with PN-1 were also analysed. Neither Smad2- and Smad3-, nor their receptor TGFβR1 (ALK5) expression was modified by PN-1 deficiency in normal conditions or 3 days post-ischemia (See Supplementary Fig. S1 online).

Because the expression of many genes is regulated by hypoxia via the expression of the major actor of ischemia, HIF-1α (hypoxia inducible factor), we compared HIF-1α expression in PN-1-deficient and WT mice. As expected, HIF-1α expression was greatly increased following ischemia (25-fold at day 3), but mRNA levels were similar in WT and PN-1^−/−^ muscles both in normal and ischemic conditions (See Supplementary Fig. S1 online).

### Leukocyte recruitment is enhanced in PN-1-deficient ischemic muscles

**Figure 6:**
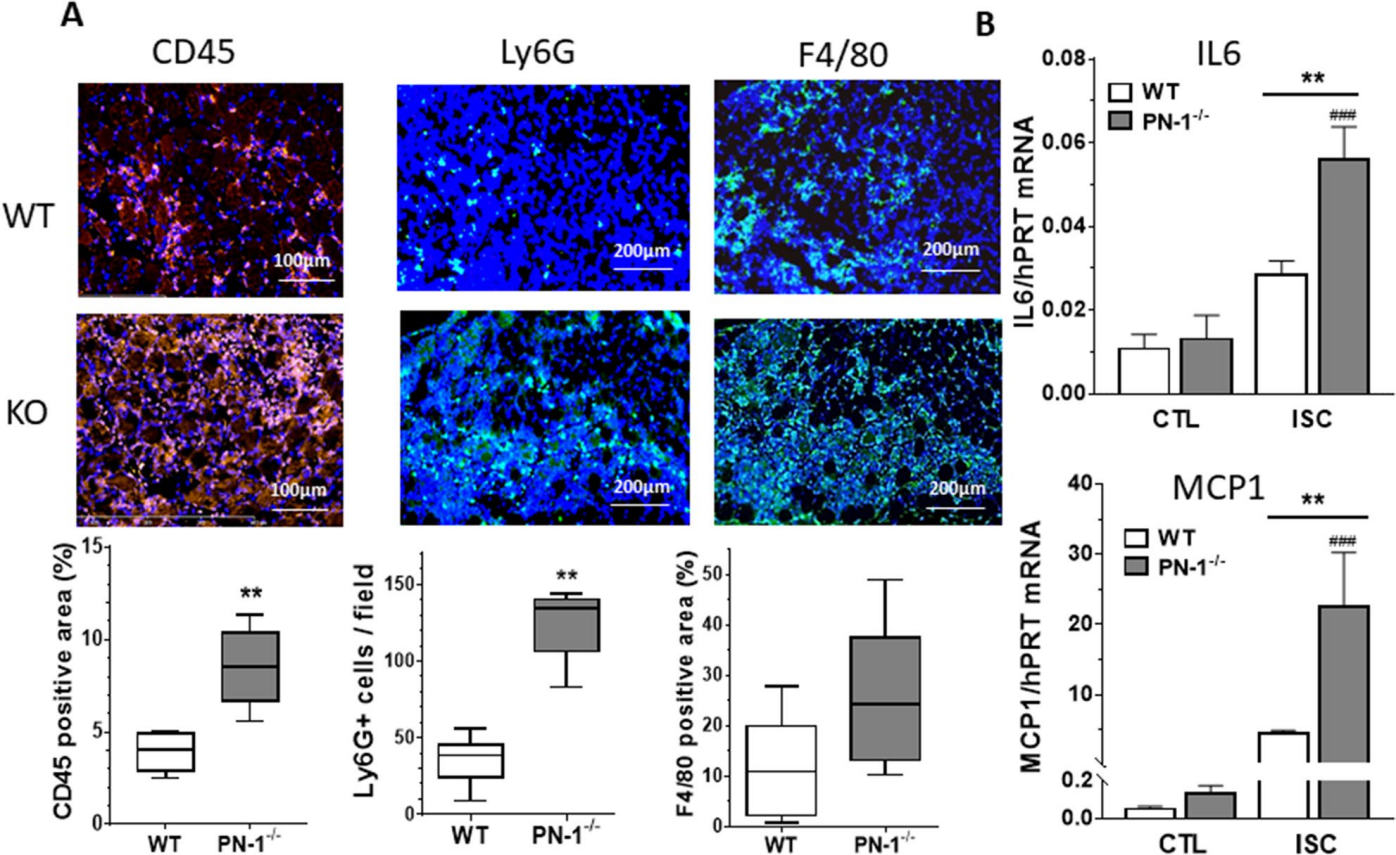
PN-1 deficiency results in higher leucocyte infiltration. Leukocytes, neutrophils and macrophages were visualized by immunostaining of ischemic muscles from WT and PN-1^−/−^ mice, 3 days after surgery, with antibodies to CD45, Ly6G and F4/80, respectively (green), and nuclei were stained with DAPI. Representative images are shown (n = 6 mice of each group). Ly6G positive cells per field were manually counted and CD45 and F4/80 positive areas were quantified as the percentage of total area using Image J software. MannWhitney **p < 0.01; for F4/80: *p* = 0,1775. * KO versus WT and # ischemic versus control.

Because MK has been shown to promote leukocyte recruitment in this model^15^, we analysed by immunostaining leukocyte enrolment in muscle sections, 3 days after surgery. Our results indicated that the influx of leukocytes (CD45) following ischemia was significantly higher in PN-1-deficient mice compared with WT mice (Fig. 6A). Labelling of macrophages (F4/80) and neutrophils (Ly6G) revealed a trend to higher macrophage recruitment and a significantly higher neutrophil recruitment in PN-1^−/−^ versus WT mice.

In agreement with these data and with MK overexpression, the mRNA levels of the pro-inflammatory IL6 cytokine and of the MCP-1 chemokine were significantly higher in PN-1^−/−^- compared to WT- ischemic muscles (Fig. 6B).

Taken together, our results indicate that the higher expression of MK observed in PN-1deficient mice is associated with an increase in leukocyte recruitment in response to higher levels of MCP-1, finally driving neoangiogenesis.

## Discussion

Neovascularization in response to ischemia is a critical adaptive phenomenon for the preservation of tissue integrity, mainly by angiogenesis and arteriogenesis, but also by collateral growth and vasculogenesis. In this study, we investigated the role of the anti-angiogenic serpin PN-1 in the regulation of the post-ischemic angiogenic response in the mouse model of hindlimb ischemia. In this model, single ligation of the femoral artery causes chronic ischemia, resulting in necrosis and apoptosis of a large part of the tissue downstream of the ligation. This phase is followed by an inflammatory phase, and finally by neovascularization and remodelling processes. Neovascularization ensures perfusion of the tissue close to the arterial occlusion and restores that of muscle tissues located distal from the occlusion^19^. In this study, we clearly show by angiographic analysis that PN-1 deficiency favours a faster and more extensive reperfusion of the muscles after ischemia. Indeed, we observed an increase in the density of reperfused vessels 3 days post-ligature. The significant higher capillary density observed in PN-1-deficient- compared to wild type ischemic muscles confirm the key role of PN-1 in post-ischemic angiogenesis. Moreover, the greater number of arterioles in PN-1^−/−^ versus WT muscles in non ischemic muscles could also favour the arteriogenic remodelling of collateral vessels following femoral artery ligation, therefore leading to faster blood flow recovery. This indicates that PN-1-deficiency permitted enhanced perfusion to the threatened ischemic regions via both greater capillary sprouting and increased arteriogenesis. Thus, PN-1 regulates not only vascular development in physiological conditions^11^, but is also involved in post-ischemic neovascularization.

Such a role of PN-1 can be explained by its expression in the numerous cells of the muscle tissue. Indeed, PN-1 expression has been demonstrated in human myotubes^20^, endothelial cells^21,22^, smooth muscle cells^23^, pericytes^24^, fibroblasts^25^, neurons^26^, and glial cells^27,28^. In the gastrocnemius muscle, we indeed detected PN-1 in nerve fibres, arteries and veins, but also in muscle fibres. The importance of PN-1 expression in muscles has also been shown in muscle differentiation^29^ and in the maintenance of neuromuscular synapses^30,31^. During the regeneration phase, we observed that the newly formed muscle fibres also express PN-1. The production of PN-1 by these fibres could therefore contribute to regulate the formation of the capillaries progressively surrounding muscle fibres. Angiogenesis requires sustained endothelial cell migration and proliferation. The anti-angiogenic activity of PN-1 can thus be explained in part by its ability to inhibit endothelial cell proliferation^12^, illustrated in the present study by the greatly increased amount of Ki67/VE-cadherin double positive cells observed in PN-1^−/−^ versus WT ischemic muscles. We previously showed in vitro that the antiprotease activity of PN-1 is not involved in the inhibition of cell proliferation. However, PN-1 target proteases like urokinase and tPA (tissue type plasminogen activator) have been involved in post-ischemic angiogenesis. Deficiency of both proteases was indeed associated with decreased angiogenesis and blood flow recovery^32,33^, and uPA gene therapy induced functionally significant angiogenesis in hind limb ischemia model^34^. Decreased inhibition of these proteases in PN-1 deficient mice could thus also account for the higher post ischemic neovascularisation observed.

The PN-1 expression profile following ischemia corresponds to that of anti-angiogenic factors (factors increasing beyond 3 days), according to the analysis of Lee et al.^35^. Indeed, in Lee’s study, genes (pro-angiogenic, pro inflammatory, stress, anti-angiogenic, anti-inflammatory and remodelling genes) were classified according to their dominant function and their expression kinetics. Our results indicate that the expression kinetics of PN-1 are delayed compared to those of pro-angiogenic factors, but fit with those of anti-angiogenic factors expressed during ischemia. PN-1 expression strongly increased during the first week following ischemia and then gradually decreased both at the protein and mRNA levels, indicating an upregulation of PN-1 in ischemic conditions, which could impair angiogenesis. The early increase in PN-1 at the protein level at the time of leukocyte recruitment, preceding the increase at the messenger level, can be explained by the fact that in the first days after ligation, PN-1 is mainly present in the recruited leukocytes. Indeed, inflammatory cells are known to express PN-1, especially in stressful situations^7^. Our results show that PN-1 deficiency favours inflammatory cell recruitment during hindlimb ischemia similarly to what was shown during lung inflammation^36^. Thus, PN-1-deficiency allowed enhanced perfusion not only via increased endothelial cell proliferation but also via enhanced leukocyte recruitment that is known to be strongly involved in collateralization^37,38^. Such a massive leukocyte recruitment observed in PN-1^−/−^ ischemic muscles can be related to greater overexpression of IL-6 and MCP-1 compared to WT ischemic muscles. Indeed, both the chemokine MCP-1 and the pro-inflammatory cytokine IL-6 trigger leukocyte infiltration and are involved in the induction of angiogenesis and arteriogenesis^39^**.** Our results show that the modulation of MK expression could explain the higher levels of MCP-1 and IL-6 in PN-1^−/−^ ischemic muscles compared to WT. Indeed, MK is known to modulate the expression of various pro-inflammatory cytokines including MCP-1 and IL-6^40,41^. We observed that the ischemia-induced overexpression of MK was enhanced in the absence of PN-1, both at the mRNA and protein levels. This agrees with previous data showing, in the postnatal retinal angiogenesis model, that hypervascularization in PN-1-deficient retina was associated with MK overexpression^11^. MK is a pro-angiogenic cytokine, belonging to the family of heparin-binding growth factors, which promotes tumour angiogenesis^42^ and angiogenesis in response to ischemia. Importantly, MK-deficient mice exhibit an almost complete defect in the angiogenic response in the hindlimb ischemia model^15^. The sustained effect of PN-1 on the expression of MK lasted throughout the neovascularization phase. MK expression could thus be modulated by PN-1 produced at a basal state in muscle as well as PN-1 induced during ischemia and/or expressed by leucocytes. Hypoxia was shown to increase MK protein expression in human polymorphonuclear neutrophils (PMN), monocytes, and human umbilical vein endothelial cells compared with normoxia^15^. However, in our study, the expression levels of HIF-1α (Hypoxia-inducible factor 1-alpha), one of the first proteins overexpressed during ischemia, were similar in PN-1-deficient and WT mice, although this hypoxia-mediated nuclear transcription factor controls the expression of many angiogenic factors including MK^43^. Thus, the effect of PN-1 on MK expression is independent of HIF-1α and involves a mechanism that remains to be deciphered. Whether MK is a direct downstream target of PN-1 remains to be determined. Indeed, PN-1 could either modify MK expression by a yet unknown mechanism or act on an intermediate target, through its antiprotease activity or not.

Taken together, our results demonstrate that PN-1 can limit neovascularisation in pathological conditions, including post-ischemic reperfusion of the lower limbs. The higher expression of MK observed in PN-1- deficient mice is associated with an increase in leukocyte recruitment in response to higher levels of MCP-1, finally driving neoangiogenesis. PN-1 is thus an important player in both physiological and pathological angiogenesis, potentially of interest to target for the modulation of angiogenic responses.

## Materials and methods

### Mice

PN-1-deficient mice^44^ and their wild-type littermate (C57BL/6 background) were generated by heterozygous mating and bred in-house. All animals were genotyped by PCR and all experiments were performed in accordance with French ethical laws. PN-1/LacZ reporter mice were generated as previously described^45^.

### Ethics

All animal experiments were performed in compliance with the institutional guidelines for the care and use of animal research and the ARRIVE guidelines. Experiments were conducted in accordance with the Animal Care and Use Committee (2011-14/69-0035) and had received approval of the French MESRI (Ministère de l’Enseignement Supérieur, de la Recherche et de l’Innovation - #8871).

### Hindlimb ischemia

To induce unilateral hindlimb ischemia, C57BL/6 male wild-type, PN-1 knockout (PN-1^−/−^), or PN-1/LacZ mice aged 8–10 weeks underwent surgical ligation of the right external iliac artery. Before surgery, mice were anesthetized by an intraperitoneal injection of a combination of ketamine (Imalgen 500, Merial, France) and xylazine (Rompun, Bayer HealthCare, France) or by isoflurane inhalation under buprenorphine analgesia. The ligation was performed on the proximal part of external iliac artery, below the bifurcation of the internal iliac artery, as described previously. The femoral vein and nerve were preserved. Analysis of post-ischemic neovascularization was evaluated by immunohistochemistry and angiography, as described previously^46,47^.

### Angiography

Three days after ligation, mice (n ≥ 8 per group) were anesthetized by a ketamine and xylazine mixture. About 20 min before angiography, mice received an intraorbital injection of 5U heparin to prevent clot formation. Then, a longitudinal laparotomy was performed to introduce a needle (30 G) attached to a polyethylene catheter into the abdominal aorta for injection of a contrast medium (barium sulphate, 1 g/mL, Micropaque, Guerbet, France) as described previously^48^. Angiography of hindlimbs was performed and images acquired with the use of an image plate system for digital radiography (VistaScan and DBSWIN Imaging Software, Dürr Dental, Germany).

Neovascularization was quantified by measuring the number of pixels occupied by vessels in a defined area with the use of ImageJ software. The area of quantification was delineated by the femoral artery ligature, the knee, the edge of the femur, and the external limit of the leg. The results were then expressed as a ratio of the ischemic to the non-ischemic leg (angiographic score).

### Immunohistochemistry: capillary density, arterioles, leucocytes and proliferation

At different times after ischemia, the calf muscles were harvested, enrobed in Tissue-Tek OCT Compound (Sakura Finetek USA, CA, USA), frozen in isopentane and then cryosectioned transversely (10 µm). Sections were fixed in 4% PFA and stained with isolectin B4-alexa fluor 568 (Invitrogen) or alternatively with a polyclonal anti VE-Cadherin (R&D systems) followed by Fab anti-rabbit IgG-Alexa fluor 568 conjugate (Invitrogen) to identify capillaries, and with a monoclonal antibody to alpha smooth muscle actin-Cy3 conjugate (Sigma) to identify arterioles. Capillary density was quantified by measuring capillary area in the gastrocnemius using Image J software, and arterioles were counted manually on high magnification fields (X200) (at least 3 sections/muscle, at least 6 mice of each group). Leukocytes were labelled using a monoclonal rat antibody to mouse CD45 (3F11, Biolegend), macrophages and neutrophils by monoclonal rat antibodies to F4/80 (A3:1, AbdSerotec) and to Ly6G (1AB, BD Pharmingen), respectively, followed by Fab anti-rat IgG-Alexa fluor 568 conjugate. Proliferation was assessed using an antibody to Ki67- Alexa fluor 488 conjugate (D3B5, rabbit Mab, Cell signaling). Midkine was detected with a polyclonal rabbit anti-mouse antibody (Bioworld BS6038). Ki67 and Ly6G Positive nuclei were counted manually and expressed as a percentage of total nuclei. CD45, MK and F4/80 expression were quantified by densitometric analysis using image J.

### RNA isolation, reverse transcription and quantitative real-time PCR

Total RNA was extracted from the tibialis muscles after crushing and homogenization, using Trizol Reagent (Invitrogen) according to the manufacturer’s protocol, and 1 µg of RNA was reverse-transcribed into cDNA using Maxima Reverse Transcriptase (ThermoScientific). Real-time PCR analysis was performed on cDNA with SYBR Green PCR Master Mix Plus (Applied Biosystems) and using the primers listed in supplementary material (Table I). The level of expression of the target gene was normalized to that of GAPDH or HPRT in each sample.

### Protein analysis

Anterior tibial muscles were harvested and frozen in liquid nitrogen. Muscle samples were crushed and homogenized in RIPA lysis buffer (50 mM Tris, pH 7.4, 150 mM NaCl, 1% NP40, 1% sodium deoxycholate, 0.1% sodium dodecyl sulphate) containing a cocktail of protease inhibitors (Sigma), briefly sonicated, and centrifuged at 10.000* g* at 4 °C. Total protein concentration was measured by BCA protein assay (Pierce). Equal amounts of protein were separated by SDS-PAGE. Samples (15 µg) were electrophoresed in 4–20% polyacrylamide gel (Pierce), and electrotransferred to a nitrocellulose membrane. Immunoblotting was performed with anti PN-1 monoclonal 4B3 (Santa Cruz), and anti-mouse secondary antibodies-HRP conjugate (Jackson).

### XGal staining

For X-Gal staining, gastrocnemius sections from PN-1/LacZ mice were fixed for 5 min in 0.5% glutaraldehyde and incubated at 37 °C with X-Gal staining solution (5 mM potassium ferricyanide, 5 mM potassium ferrocyanide, 2 mM MgCl2, 1 mg/ml X-Gal in PBS).

### Statistical analysis

Data are expressed as means ± SEM. Statistical analysis of differences between groups was performed with the GraphPad Prism software. The Shapiro–Wilk test was used to analyse the data distribution, and thus determine the appropriate test as indicated. A *p* value less than 0.05 was considered significant.

## Supplementary Information

Supplementary Information.

## Data Availability

The datasets generated during and/or analysed during the current study are available from the corresponding author on reasonable request.

## References

[1] Inampudi C, Akintoye E, Ando T, Briasoulis A (2018). Angiogenesis in peripheral arterial disease. Curr. Opin. Pharmacol..

[2] Ikeda Y (2012). Heparin cofactor II, a serine protease inhibitor, promotes angiogenesis via activation of the AMP-activated protein kinase-endothelial nitric-oxide synthase signaling pathway. J. Biol. Chem..

[3] Miao RQ, Agata J, Chao L, Chao J (2002). Kallistatin is a new inhibitor of angiogenesis and tumor growth. Blood.

[4] Tashiro Y (2012). Inhibition of PAI-1 induces neutrophil-driven neoangiogenesis and promotes tissue regeneration via production of angiocrine factors in mice. Blood.

[5] Boulaftali Y (2010). Anticoagulant and antithrombotic properties of platelet protease nexin-1. Blood.

[6] Bouton MC (2012). Emerging role of serpinE2/protease nexin-1 in hemostasis and vascular biology. Blood.

[7] Mansilla S (2008). Macrophages and platelets are the major source of protease nexin-1 in human atherosclerotic plaque. Arterioscler. Thromb. Vasc. Biol..

[8] Moller MJ, Qin Z, Toursarkissian B (2012). Tissue markers in human atherosclerotic carotid artery plaque. Ann. Vasc. Surg..

[9] Strehlow D, Jelaska A, Strehlow K, Korn JH (1999). A potential role for protease nexin 1 overexpression in the pathogenesis of scleroderma. J. Clin. Investig..

[10] Francois D (2014). Increased expression of protease nexin-1 in fibroblasts during idiopathic pulmonary fibrosis regulates thrombin activity and fibronectin expression. Lab. Investig..

[11] Selbonne S (2015). Protease nexin-1 regulates retinal vascular development. Cell. Mol. Life Sci..

[12] Selbonne S (2012). In vitro and in vivo antiangiogenic properties of the serpin protease nexin-1. Mol. Cell. Biol..

[13] Cai YQ (2020). Multiple pathophysiological roles of midkine in human disease. Cytokine.

[14] Shin DH (2020). Midkine is a potential therapeutic target of tumorigenesis, angiogenesis, and metastasis in non-small cell lung cancer. Cancers (Basel).

[15] Weckbach LT (2012). Midkine acts as proangiogenic cytokine in hypoxia-induced angiogenesis. Am. J. Physiol. Heart Circ. Physiol..

[16] Weckbach LT (2014). The cytokine midkine supports neutrophil trafficking during acute inflammation by promoting adhesion via beta2 integrins (CD11/CD18). Blood.

[17] Scholz D (2002). Contribution of arteriogenesis and angiogenesis to postocclusive hindlimb perfusion in mice. J. Mol. Cell. Cardiol..

[18] Schmidt CA (2018). Strain-dependent variation in acute ischemic muscle injury. Am. J. Pathol..

[19] Aref Z, de Vries MR, Quax PHA (2019). Variations in surgical procedures for inducing hind limb ischemia in mice and the impact of these variations on neovascularization assessment. Int. J. Mol. Sci..

[20] Mbebi C, Hantai D, Jandrot-Perrus M, Doyennette MA, Verdiere-Sahuque M (1999). Protease nexin I expression is up-regulated in human skeletal muscle by injury-related factors. J. Cell. Physiol..

[21] Bouton MC (2007). Protease nexin-1 interacts with thrombomodulin and modulates its anticoagulant effect. Circ. Res..

[22] Leroy-Viard K, Jandrot-Perrus M, Tobelem G, Guillin MC (1989). Covalent binding of human thrombin to a human endothelial cell-associated protein. Exp. Cell Res..

[23] Bouton MC (2003). The serpin protease-nexin 1 is present in rat aortic smooth muscle cells and is upregulated in L-NAME hypertensive rats. Arterioscler. Thromb. Vasc. Biol..

[24] Kim JA (2006). Brain endothelial hemostasis regulation by pericytes. J. Cereb. Blood Flow Metab..

[25] Baker JB, Low DA, Simmer RL, Cunningham DD (1980). Protease-nexin: a cellular component that links thrombin and plasminogen activator and mediates their binding to cells. Cell.

[26] Reinhard E, Suidan HS, Pavlik A, Monard D (1994). Glia-derived nexin/protease nexin-1 is expressed by a subset of neurons in the rat brain. J. Neurosci. Res..

[27] Cunningham DD (1992). Regulation of neuronal cells and astrocytes by protease nexin-1 and thrombin. Ann. N. Y. Acad. Sci..

[28] Monard D (2017). SERPINE2/Protease Nexin-1 in vivo multiple functions: Does the puzzle make sense?. Semin. Cell Dev. Biol..

[29] Verdiere-Sahuque M (1996). Myoblast fusion promotes the appearance of active protease nexin I on human muscle cell surfaces. Exp. Cell Res..

[30] Akaaboune M (1998). Developmental regulation of the serpin, protease nexin I, localization during activity-dependent polyneuronal synapse elimination in mouse skeletal muscle. J. Comp. Neurol..

[31] Festoff BW, Rao JS, Hantai D (1991). Plasminogen activators and inhibitors in the neuromuscular system: III. The serpin protease nexin I is synthesized by muscle and localized at neuromuscular synapses. J. Cell. Physiol..

[32] Deindl E (2003). Receptor-independent role of the urokinase-type plasminogen activator during arteriogenesis. FASEB J..

[33] Leu S (2014). Retention of endothelial progenitor cells in bone marrow in a murine model of endogenous tissue plasminogen activator (tPA) deficiency in response to critical limb ischemia. Int. J. Cardiol..

[34] Traktuev DO (2007). Urokinase gene transfer augments angiogenesis in ischemic skeletal and myocardial muscle. Mol. Ther..

[35] Lee CW (2004). Temporal patterns of gene expression after acute hindlimb ischemia in mice: insights into the genomic program for collateral vessel development. J. Am. Coll. Cardiol..

[36] Francois D (2018). Hematopoietic protease nexin-1 protects against lung injury by preventing thrombin signaling in mice. Blood Adv..

[37] Fung E, Helisch A (2012). Macrophages in collateral arteriogenesis. Front. Physiol..

[38] Troidl C (2013). The temporal and spatial distribution of macrophage subpopulations during arteriogenesis. Curr. Vasc. Pharmacol..

[39] Shireman PK (2007). The chemokine system in arteriogenesis and hind limb ischemia. J. Vasc. Surg..

[40] Sato W (2002). Midkine expression in the course of nephrogenesis and its role in ischaemic reperfusion injury. Nephrol. Dial. Transplant..

[41] Shindo E (2017). The growth factor midkine may play a pathophysiological role in rheumatoid arthritis. Mod. Rheumatol..

[42] Ota T (2010). Midkine expression in malignant salivary gland tumors and its role in tumor angiogenesis. Oral Oncol..

[43] Rey S, Semenza GL (2010). Hypoxia-inducible factor-1-dependent mechanisms of vascularization and vascular remodelling. Cardiovasc. Res..

[44] Luthi A (1997). Endogenous serine protease inhibitor modulates epileptic activity and hippocampal long-term potentiation. J. Neurosci..

[45] Kvajo M (2004). Regulation of brain proteolytic activity is necessary for the in vivo function of NMDA receptors. J. Neurosci..

[46] Hellingman AA (2010). Variations in surgical procedures for hind limb ischaemia mouse models result in differences in collateral formation. Eur. J. Vasc. Endovasc. Surg..

[47] Silvestre JS (2003). Vascular endothelial growth factor-B promotes in vivo angiogenesis. Circ. Res..

[48] Loinard C (2009). Inhibition of prolyl hydroxylase domain proteins promotes therapeutic revascularization. Circulation.

