# Supplementary material for: Protease nexin-1 deficiency increases mouse hindlimb neovascularisation following ischemia and accelerates femoral artery perfusion
Source: Sci Rep. 2021 Jun 28;11:13412. doi: 10.1038/s41598-021-92794-9 (PMC8238971; doi:10.1038/s41598-021-92794-9)
Supplement: Supplementary file 1 — Supplementary Information. [file 41598_2021_92794_MOESM1_ESM.pdf]

Supplemental information

Protease nexin-1 deficiency increases mouse hindlimb neovascularisation following ischemia and accelerates femoral artery perfusion

Sonia Selbonne[1], Celina Madjene[1], Benjamin Salmon[2], Yacine Boulaftali[1], Marie-Christine Bouton[1], Véronique Arocas[1]*.

[1]LVTS, INSERM, U1148, Paris, France ; Université de Paris, Paris, France.

[2] EA 2496 Pathologies Imagerie et Biothérapies de l'organe dentaire, Université de Paris, Faculté de Chirurgie Dentaire, Montrouge – France

* Corresponding author: Dr Véronique AROCAS
Unité INSERM U1148, CHU Xavier Bichat
46 rue Henri Huchard 75877 Paris Cedex 18, France
Tel: 33 (1) 40.25.75.32
Fax: 33 (1) 40.25.86.02
E-mail: veronique.arocas@inserm.fr

Sup Fig S1: Expression levels of Smad2, Smad3, TGFβR1 and HIF-1α in PN-1-deficient and wild-type muscles in control conditions and 3 days following ischemia.

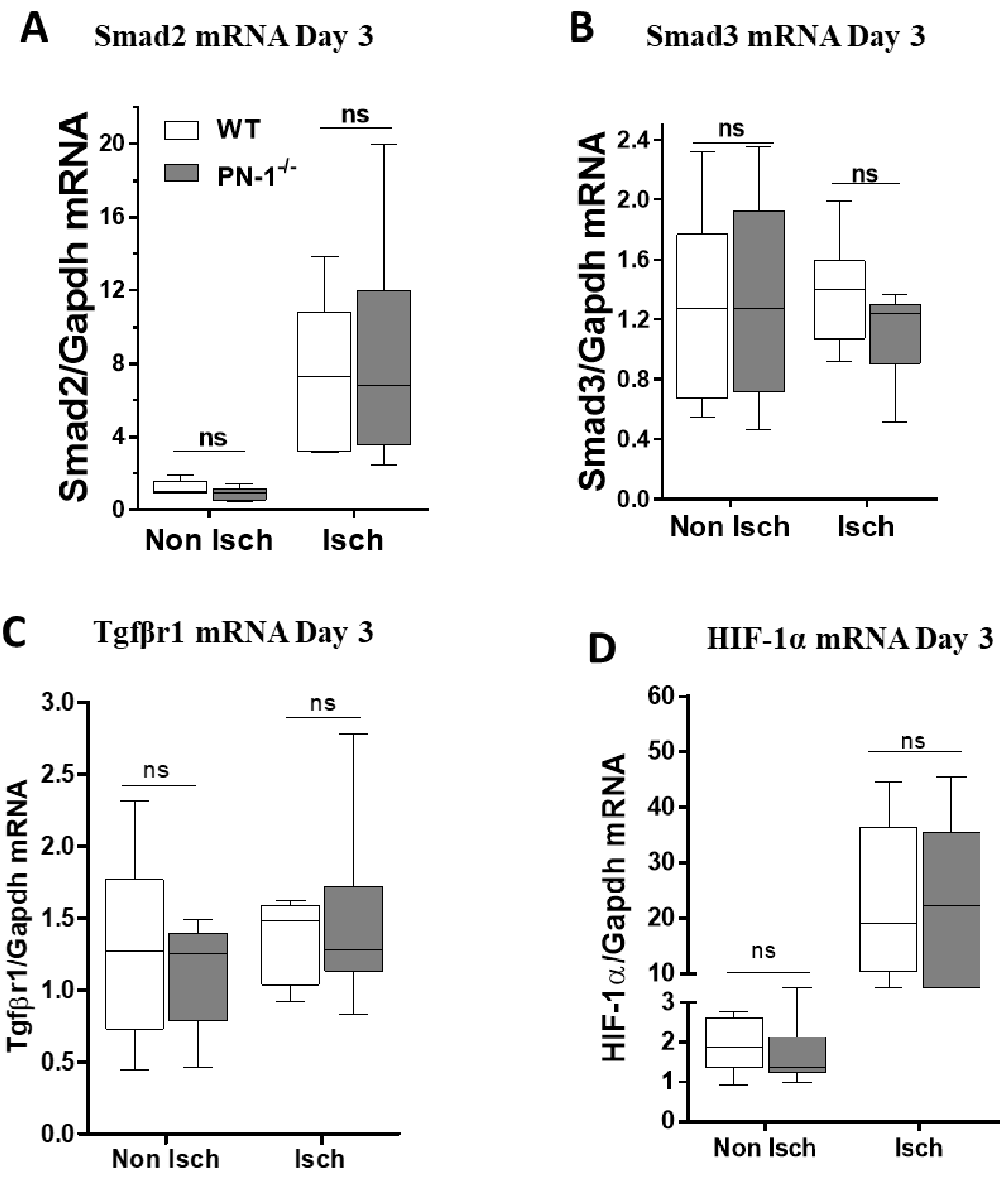

Sup Fig S2: Uncropped image of Fig 1B: immunoblotting of muscle lysates with antibody to PN-1 and densitometric analysis.

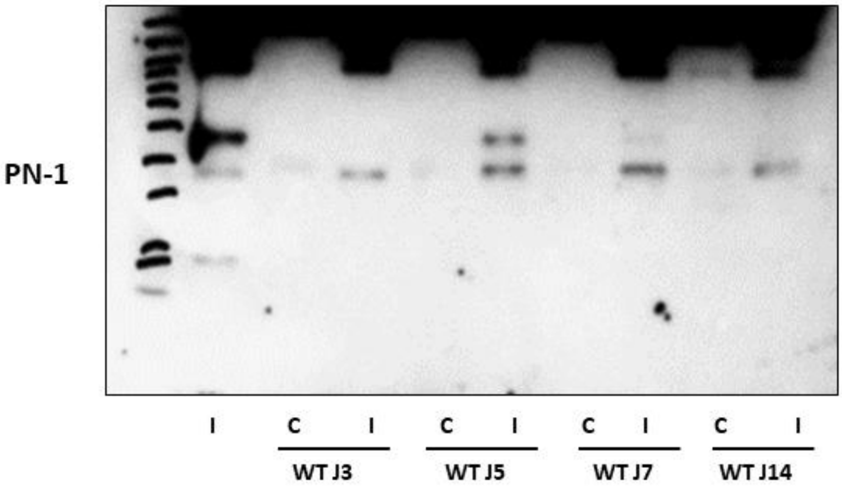

Supplemental Table 1: Primer sequences (5'-3') and PCR conditions for RT-qPCR analysis.

GenBank Reference No.	Gene Abbreviation	Primers (Forward, reverse)	Annealing T° (C°)	Product size
	PN-1	cagtgtgaagtgcagaatgtga<br>ttggggaaagcagattatcaa	60	112
NM_013556.2	HPRT	tcctcctcagaccgctttt<br>cctggttcatcatcgctaatc	60	90
NM_031168.2	IL-6	ccagttgccttcttgggact<br>ggtctgttgggagtggtatcc	60	101
NM_011333.3	MCP1	tcactgaagccagctctctct<br>gtggggcgttaactgcat	66	127
NM_010784.4	MDK	cacctccaagaccaagtcaaa<br>caaaaggcactggtgggtta	65	148
NM_010431.2	HIF-1α	gcattgtgtgtgtgaattatgttg<br>tgaacagctgagtcatcttcatatc	65	126
NM_009370.2	Tgfβr1	ctagagaagagcgttcatggttc<br>cgtccatgtcccattgtct	66	116
NM_008541.3	Smad5	gcagtaacatgattcctcagacc<br>gcgacaggctgaacatctct	66	61
NM_010754.5	Smad2	tgccactgtagaaatgacaagaa<br>cactatcacttaggcactcagca	65	95
NM_016769.4	Smad3	tccgtatgagcttcgtcaaa<br>ggtgctggtcactgtctgtc	65	62
NM_008084.2	Gapdh	tgtccgtcgtggatctgac<br>cctgcttcaccaccttcttg	65	74
